# Editorial: Phytochemical-based nanoformulations to tackle drug resistance in cancer

**DOI:** 10.3389/fphar.2023.1165596

**Published:** 2023-03-16

**Authors:** Hardeep Singh Tuli, Ginpreet Kaur, Mükerrem Betül Yerer

**Affiliations:** ^1^ Department of Biotechnology, Maharishi Markandeshwar Engineering College, Maharishi Markandeshwar (Deemed to Be University), Ambala, India; ^2^ Department of Pharmacology, Shobhaben Pratapbhai Patel School of Pharmacy and Technology Management, SVKM’S NMIMS, Mumbai, India; ^3^ Faculty of Pharmacy, Department of Pharmacology, Drug Application and Research Center, Erciyes University, Kayseri, Türkiye

**Keywords:** chemoprevention, phytochemicals, nanotherapeutic, drug resistance, cell signaling

A significant problem in medical oncology is chemoresistance. Resistance developed during treatments is a major cause of fatalities in cancer patients. Conventional chemotherapeutic agents have also been revealed to cause damage to normal cells in addition to resistance, suggesting that treatment interruption may be necessary. Phytochemicals might protect healthy tissues *via* their antioxidant activities and can avoid the emergence of resistance. Their low solubility and short half-life, however, limit their use. Currently, phytonanotechnology is being developed to offer the advantages of increased solubility and availability of phytochemicals. Phytochemicals can reach cancer cells after being attached to nanovehicle surfaces or combined with substances that target tumors. Micelles, liposomes, and nanoparticles are examples of nanoformulations that have the potential to be highly loaded with phytochemicals, including weakly soluble and poorly stable phytochemicals. It will boost the phytochemical bioavailability to local tumors and lower the chances of unfavorable side effects in neighboring or distant organs.

Singh et al. offer an insightful overview of clinical updates on tyrosine kinase inhibitors in HER2-positive breast cancer, with a focus on therapeutic advancements such as the use of nanotechnological interventions. To overcome drug resistance and limited efficacy in current treatment options, nanoformulations can be used in patients receiving HER2^+^ BC treatment. Anti-HER2 ligands can be used in various nanoformulations to target HER2 receptors. Here, the authors discuss targeted TKIs in patients with HER2^+^ BC, clinical studies of HER2^+^-targeted TKIs, mechanisms of resistance to HER2-directed therapies with new implications of TKIs in HER2^+^ MBC (metastatic breast cancer), and anti-HER2 ligands in various nanoformulations to target HER2 receptors.

Another interesting advancement is the popularization of network pharmacology as an approach to screening new therapeutic targets and moieties. The research work shared by Ahmed et al. sheds light on the exploration of MARK4 as a target for the management of hepatocellular carcinoma and establishes the role of phytoconstituents in regulating the expression of proteins and interactions with altered genes.

An intriguing study by Upadhyay et al. focuses on the therapeutic potentials of yeast-derived *β*-1,3-glucan particles as a safe and effective approach to ameliorating cervical cancer. A need to explore alternative therapeutic strategies to improve therapeutic outcomes and the quality of life of patients has been identified. Using instrumental techniques, the authors characterize the particles and establish an effective dosage. The authors also highlight the need for further research in order to develop a deeper understanding of the spectrum of actions and potentials for the clinical use of the agent studied.

The review article by Sindhoor et al. investigates the role of alkaloid nanoformulations for lung cancer therapy as an approach to tackling commonly associated problems with the delivery of conventional therapeutic agents for disease management. The review explores the mechanism of different alkaloids with reference to the pathways targeted, discusses the drawbacks associated with conventional treatment strategies, and highlights the need for nanoformulations, moving forward.

Kaur et al. analyze the efficacy of Frankincense oil-loaded nanoemulsion formulation of paclitaxel and erucin, focusing on drug resistance in breast cancer. The study explores the rationale for the selection of the constituents, signaling pathways targeted, and studies that involved evaluation in an animal model to elucidate the synergistic role of the active components in overcoming drug resistance. The study offers an in-depth overview of the formulation considerations and due modulations, effects on various biomarkers, and prospects for further research and development.

Singh et al. explore the domain of Ehrlich-ascites carcinoma, proposing the development and use of nanoparticles loaded with erucin, a dietary isothiocyanate isolated from *Eruca sativa.* The authors discuss the antioxidant property of the phytoconstituent, established using a variety of *in vitro* assay techniques, and its effects on cellular proliferation. A novel approach to erucin delivery, through the designing of cubosomes, is elucidated, which shall pave the way for further advancements and applications in clinical settings. We sincerely hope you enjoy reading the articles in this Research Topic, which address a subject that is likely to become even more decisive in the coming years.

